# Data Mining Trauma: AI-Assisted Qualitative Study of Cyber Victimization on Reddit

**DOI:** 10.2196/75493

**Published:** 2025-09-03

**Authors:** J'Andra Antisdel, Wendy R Miller, Doyle Groves

**Affiliations:** 1 Center for Enhancing Quality of Life in Chronic Illness, School of Nursing, Indiana University Indianapolis, 600 Barnhill Drive, Indianapolis, IN, 46202, United States, 1 574-703-4472; 2 Center for Integrated Healthcare Education, Saint Mary's College, Notre Dame, IN, United States

**Keywords:** cyber victimization, word adjacency graphing, cyberbullying, artificial intelligence, data mining, thematic analysis

## Abstract

**Background:**

Cyber victimization exposes individuals to numerous risks. Developmental and psychological factors may leave some users unaware of the potential dangers, increasing their susceptibility to psychological distress. Despite this vulnerability, methods for identifying those at risk of cyber victimization within health care settings are limited, as is research that explores their experiences of cyber victimization. The purpose of this study was to analyze how users describe experiences of cyber victimization on the social media platform Reddit (Reddit, Inc) using data mining.

**Objective:**

This study aimed to analyze and describe how users on Reddit describe and discuss their experience of cyber victimization using data mining and computational analysis of unsolicited data.

**Methods:**

This computational qualitative study used data mining, Word Adjacency Graph (WAG) modeling, and thematic analysis to analyze discussions of Reddit users surrounding cyber victimization. Inclusion criteria included posts from 2012 to 2023 from subreddits r/cyberbullying and r/bullying. GPT-4 (OpenAI), an advanced artificial intelligence language model, summarized posts and assisted in cluster labeling. Posts were reviewed to remove irrelevant content and duplicates. User anonymity was maintained throughout the study.

**Results:**

A total of 13,381 posts from 3283 Reddit were analyzed, with approximately 5.1% (n=678) originating between 2012 and 2018 and 94.9% (n=12,703) from 2019 to 2023. The WAG modeling approach identified 38 clusters, with 35 deemed to be relevant to cyber victimization experiences. Two clusters containing irrelevant material were excluded. Six overarching themes emerged: (1) psychological impact, (2) coping and healing, (3) protecting yourself online, (4) protecting yourself offline, (5) victimization across various settings, and (6) seeking meaning and understanding.

**Conclusions:**

The study highlights the effectiveness of data mining and AI in analyzing large public datasets for qualitative research. These methods can inform future studies on risky internet behavior, victimization, and assessment strategies in health care settings.

## Introduction

Cyber victimization refers to harmful experiences that occur through the internet, social media, or communication devices. These experiences are often psychologically distressing and have been linked to depression, anxiety, self-harm, and suicidal ideation [1-5].

Although well-documented as a public health concern [6,7], there are limitations in the research, including a lack of studies exploring cyber victimization from the perspectives of those who experience it. Traditional qualitative studies have contributed valuable insights into cyber victimization experiences [8,9] but are often limited by small sample sizes and researcher bias [10]. In addition, instruments to detect cyber victimization often have different methods for operationalization, using specific terms or providing brief descriptions of acts or experiences [11]. The terminology researchers use to define cyber victimization also may not align with the individual perceptions of the experience. For example, in 1 study [12], users provided more reliable responses when the term “cyber victimization” was used rather than “cyberbullying,” suggesting that language choices influence how participants relate to research prompts.

Data mining and computational qualitative analysis, which involves using computer algorithms and software to collect and analyze qualitative data, is a novel method for understanding cyber victimization. This method has been successfully used to study various public health issues such as substance abuse [13], epilepsy [14], and intimate partner violence [15].

By mining data from social media sites, researchers can access large-scale data that reflect participants’ genuine, organic thoughts, feelings, and discussions. Analyzing this user data can reveal patterns and trends that are not apparent using traditional research methods. This information can then be used to inform the development and implementation of interventions tailored to a specific population’s unique experiences and needs.

In this study, we applied data mining and computational qualitative analysis to explore how individuals describe and discuss their experience of cyber victimization on the social media platform Reddit (Reddit, Inc). Our aim was to identify patterns and themes in unsolicited narratives. The findings of this study will inform future interventions and improve methods for identifying and supporting individuals who experience cyber victimization.

## Methods

### Study Design Overview

This qualitative computational analysis used data mining and Word Adjacency Graph (WAG) modeling [16] to examine cyber victimization narratives shared by users on Reddit. Data were collected from 2 subreddits, r/cyberbullying and r/bullying, over an 11-year period (2012‐2023) to capture relevant trends and patterns in discussions surrounding cyber victimization. A systematic data extraction process was conducted using Reddit’s application programming interface (API) and a custom web-scraping tool to gather posts and comments. After data cleaning to remove irrelevant duplicates and bot-generated content, WAG modeling was applied to identify patterns and thematic clusters within the text. Following cluster identification, GPT-4 (OpenAI) was used to generate preliminary labels and summaries, which were then manually reviewed for accuracy. A keyword searching process was also conducted to account for evolving language, slang, and abbreviations. Finally, a thematic analysis was performed to refine clusters into relevant themes.

### Study Setting and Population

Reddit is a widely used social media and news aggregate platform with over 50 million active users [17]. It is known for its anonymous membership, allowing users to share their thoughts and opinions without revealing their identity. The platform is divided into over 10,000 “subreddits” with a wide range of topics, from current events and politics to hobbies and interests [18].

Although there are approximations regarding the age demographics of Reddit [19], Reddit does not collect demographic data, making it impossible to determine the exact demographic of users. Despite this limitation, Reddit has been used in previous research studies, providing valuable insights into mental health topics [20-22]. As demographic information cannot be verified, this study uses the term “users” to describe individuals who authored posts and comments. The study targeted subreddits r/cyberbullying and r/bullying for data mining, as these communities encourage personal discussions of cyber victimization. Data extraction focused on titles, post bodies, and comments from 2012‐2023.

### Data Mining and Computational Analysis

**Figure 1.**
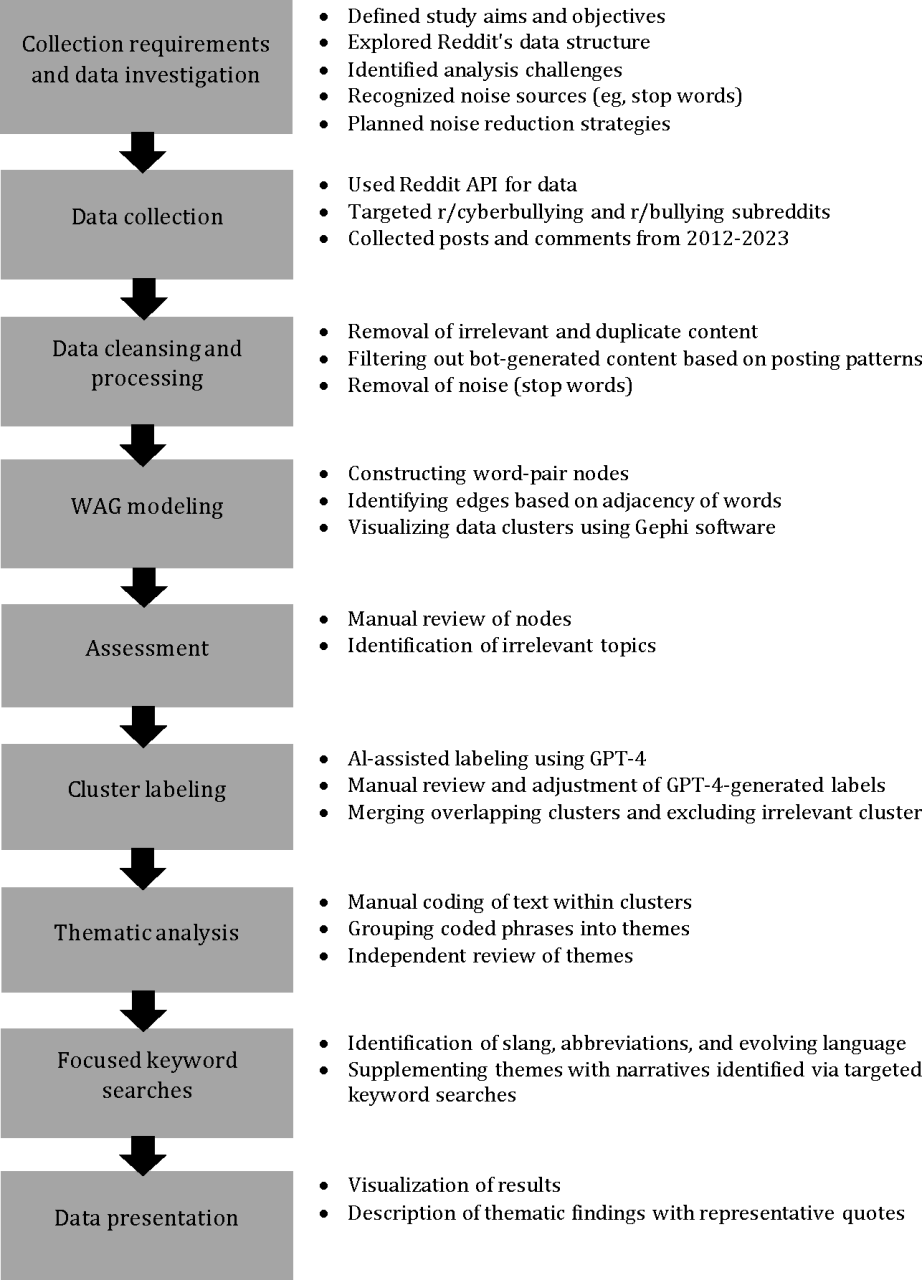
Overview of the methodological process. AI: artificial intelligence; API: application programming interface; WAG: Word Adjacency Graph;

**Figure 2.**
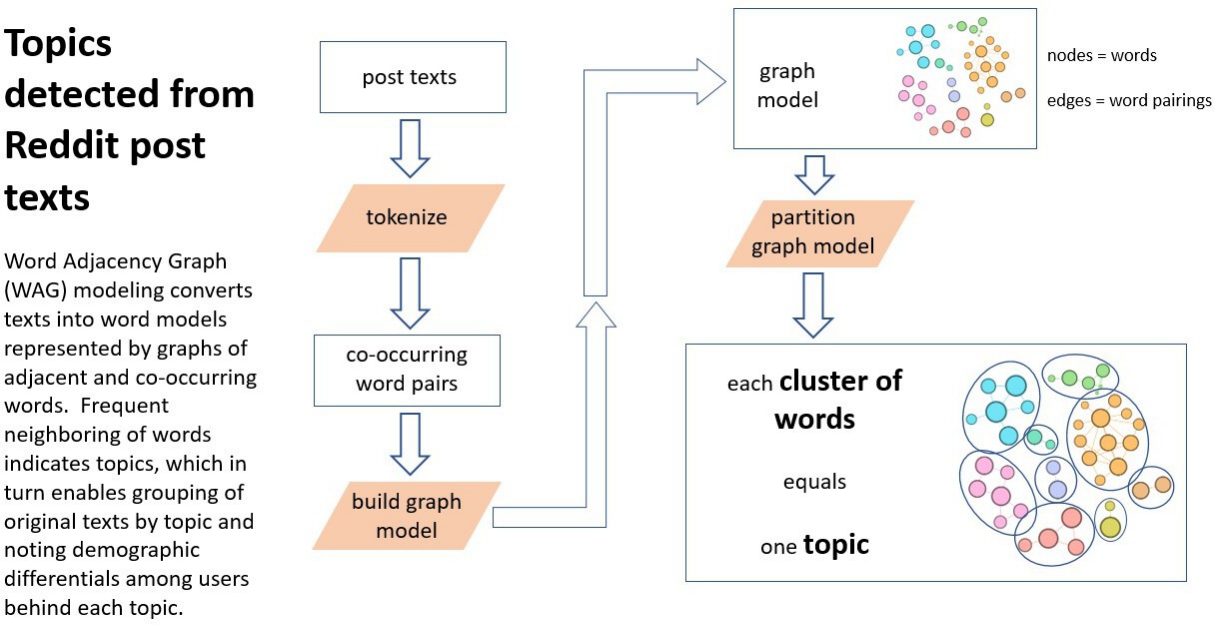
Topic detection via Word Adjacency Graph modeling.

This study used data mining and WAG modeling [16] to examine discussions on Reddit about cyber victimization. Data mining was first conducted, followed by WAG modeling, to reveal patterns and relationships between words and concepts and identify common themes within the text. Data mining consultations were conducted to enhance the validity and to confirm that the process was in alignment with the aim of the study, which was to analyze how users on Reddit describe and discuss their experience of cyber victimization. Following a systematic approach [23], data mining comprised 6 stages: collecting requirements, data investigation, data collection, modeling, assessment, and presentation [24-30]. These are outlined in Multimedia Appendix 1. A visual overview of the full methodological process is provided in Figure 1, and the topic detection process using WAG modeling is illustrated in Figure 2.

#### Thematic Analysis of Clusters

Following labeling, clusters were thematically analyzed with MAXQDA 2022 (VERBI Software GmbH), a qualitative data management software program [29], to organize clusters into overlapping themes. The full dataset was organized and prepared in a Microsoft Excel document, arranged by cluster, as well as into categories of weak clusters and those not fitting into any cluster. Each post or comment within the clusters was assigned an individual identification number. For example, when a post or comment is the 61st data point within cluster 4, it would be labeled as “C4-61.”

Posts and comments organized by cluster were transcribed into MAXQDA and coded with key phrases based on the content and context. Phrases that were similar in context were grouped and organized into themes. Following the categorization of the clusters into themes, another researcher independently reviewed these themes to ensure accuracy and consistency. This review process involved a thorough examination of how the themes were derived, ensuring that they accurately reflected the key phrases and context from the original posts and comments. The reviewer also assessed the alignment of the themes with the overall objectives of the study, adjusting where necessary to address any discrepancies or oversights.

### Ethical Considerations

This study was reviewed by the Institutional Review Board at Indiana University and determined to be exempt under category 4(i): publicly available information or specimens (Protocol #18415; initial approval February 28, 2023). The exemption was granted because the research involved analysis of publicly accessible Reddit posts without direct interaction with human subjects. Informed consent was not sought, as the data were unsolicited, publicly available, and collected in accordance with established guidelines for internet-based research on publicly accessible content without user interaction, as outlined by Eysenbach and Till [31]. To protect privacy and confidentiality, no usernames, profile information, or other potentially identifying details were stored or reported, and example quotes were paraphrased when necessary to minimize traceability via search engines. All electronic data were collected and stored on encrypted devices. Data collection complied with Reddit’s API access policies [32]. Funding for this research was made possible (in part) by Grant Number 5H79SM080386-05 from the Substance Abuse and Mental Health Services Administration (SAMHSA).

## Results

### Overview

This study successfully applied data mining, WAG modeling, GPT-4–assisted labeling, and thematic analysis to examine cyber victimization narratives shared by users on Reddit. The extracted dataset comprised 13,381 posts and comments from 3283 unique Reddit users. Approximately 5.1% (n=678) of the posts were posted between 2012 and 2018. The remaining 94.9% (n=12,703) of the posts were posted to Reddit from 2019‐2023.

**Figure 3.**
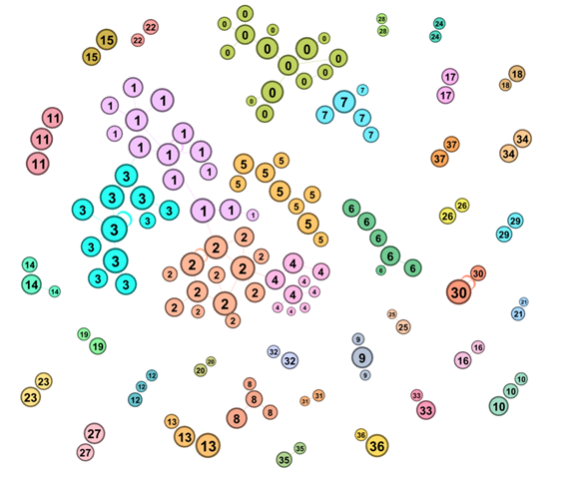
Word Adjacency Graph results cluster numbers.

**Figure 4.**
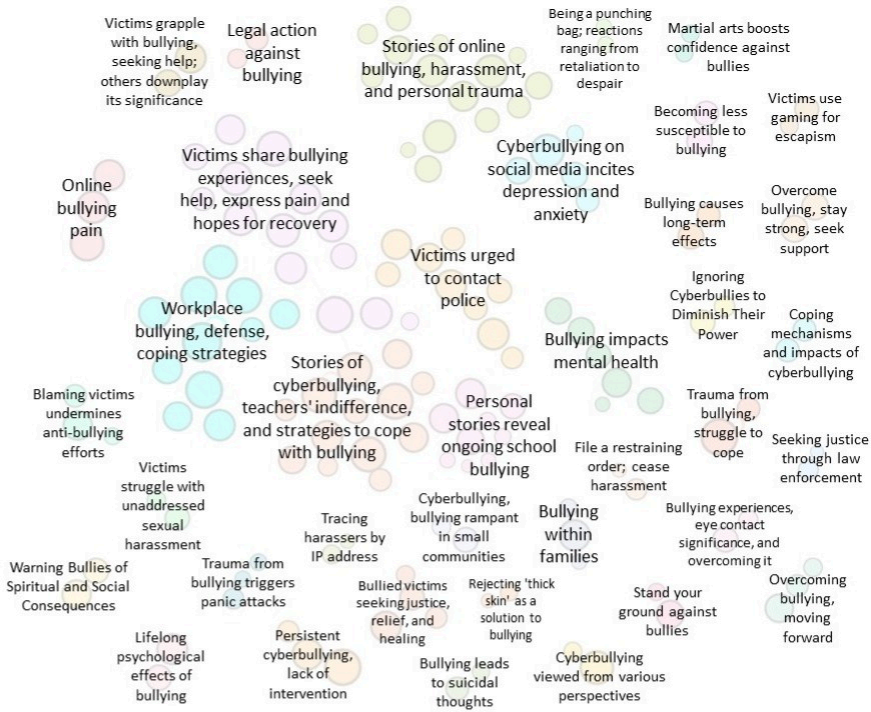
Word Adjacency Graph results cluster labels.

To construct the WAG model, only words and word pairs appearing more than 10 times were included, resulting in 150 unique words and 123 word pairs for analysis. As a result, 15% (n=1965) of posts and comments were strongly categorized, 62% (n=8290) were weakly categorized, and 23% (n=2984) were not fitted into the model. The posts that were strongly categorized formed the basis of the 38 clusters (Figure 3), which were then visualized using Gephi (Gephi Consortium). In this visualization, each node represents a word pair, with node size reflecting word frequency and proximity to similar words. Of the 38 clusters, 35 were relevant to cyber victimization experiences, while 2 clusters were excluded due to irrelevance. In addition, the WAG modeling process placed the word pairs of “law enforcement” and “legal action” into 2 clusters, but due to their overlapping content, they were merged into a single cluster (Figure 4).

The distribution of posts and comments across clusters varied, with some clusters containing a high volume of discussion while others had minimal engagement (Multimedia Appendix 2). Clusters 1 and 2 had the highest number of posts and comments, followed by clusters 3 and 0. Some clusters contained fewer than 20 posts and comments, indicating less frequent discussion of those themes. A full list of cluster labels and their thematic categorization is provided in Multimedia Appendix 3.

### Cluster Labeling and Validation

All Clusters were manually reviewed and excluded from the model in instances in which users were identified as automated bots or where posts consisted of research recruitment by researchers. The analysis involved screening for data skewness caused by the overrepresentation of individual users. This occurred in situations where users repeatedly posted similar content, which artificially inflated the presence of certain themes or topics in the dataset. Such repetitive postings, not indicative of genuine user interactions, were identified and excluded to ensure the integrity and representativeness of the data.

In analyzing online interactions and narratives surrounding cyber victimization, GPT-4 was initially used to label the resulting 38 clusters (see MultimediaAppendices 2^4^). GPT-4 synthesized a subset of posts and comments from each cluster to generate suggestive labels. The mean number of posts per cluster was 22 (SD 1) , with a typical range of 13‐30 posts; the smallest cluster included 7 posts, and the largest contained 44. These sample sizes were shaped by technical limitations at the time of the study (2023) when the GPT-4 model’s context window restricted how much data could be processed at once. At that time, approximately 819 (40%) posts of the total cluster data were used in GPT-4–assisted label generation.

Each suggested label was subsequently evaluated by a human reviewer. The manual review process relied on five validation categories: (1) retained, (2) adjusted, (3) revised, (4) merged, and (5) excluded, which were applied based on how closely the GPT-4 suggested label aligned with the cluster data. As a result, 12 labels were retained with only minimal word changes, 12 labels were adjusted, meaning the suggested label generally matched the content but required rewording to improve clarity or reflect the data more accurately, and 10 labels were completely revised to align with the content of the posts. Two clusters were merged due to thematic overlap, and 2 were excluded from the analysis due to irrelevance (see Multimedia Appendix 4).

To enhance interpretive rigor, a secondary researcher reviewed all cluster labels and confirmed their alignment with the underlying post content and resulting themes. While formal interrater reliability metrics were not calculated, consensus was achieved through collaborative review and discussion.

Overall, GPT-4 provided a usable starting point for 36 out of 38 clusters (94.7%), with 26 clusters (68%) requiring some degree of human refinement. Definitions and illustrative examples of each validation category are provided in Multimedia Multimedia Appendix 5.

### Focused Keyword Searching

Focused keyword searching was used to expand the analysis of clustered data and improve the identification of themes. This approach involved reviewing the narratives within each cluster to identify key concepts, expressions, or themes. From these, related terms and alternative phrasings were generated and used as targeted search terms across the dataset. For example, a narrative discussing a specific form of cyberbullying, like blackmail, would lead to the identification of related terms such as “blackmail” and “catfish” (Multimedia Appendix 6). This strategy allowed for the identification of narratives that were weakly clustered or not clustered at all due to variations in words or phrasing. The total number of posts retrieved via focused keyword searching was not tracked. However, the process did lead to the identification of additional narratives that were thematically aligned but not strongly categorized. These supplemental posts helped confirm existing themes and broader representation of experiences.

### Thematic Analysis

Following labeling, clusters were thematically analyzed with MAXQDA 2022, a qualitative data management software program [29], to organize clusters into overlapping themes. Posts and comments organized by cluster were transcribed into MAXQDA and read and coded with key phrases based on the content and context. Phrases that were similar in context were grouped and organized into themes.

As a result of this analysis, six themes emerged: (1) psychological impact, which examines the symptoms of cyber victimization; (2) coping and healing, focusing on healing and overcoming cyber victimization and seeking support; (3) protecting yourself online, highlighting methods for preventing or stopping cyber victimization; (4) protecting yourself offline, detailing methods to decrease the risk of being targeted in the physical world; (5) victimization across various settings, exploring the dynamics of victimization in different environments; and (6) seeking meaning and understanding, which includes philosophical discussions about the nature of victimization. A summary table outlining each theme with supporting subtopics is provided in Multimedia Appendix 7. A detailed thematic analysis of the qualitative findings will be presented in a separate publication where the themes will be explored in-depth, along with direct quotes and case examples.

## Discussion

### Principal Findings

This study identified 6 overarching themes in Reddit posts related to cyber victimization: psychological impact, coping and healing, protecting oneself online, protecting oneself offline, victimization across various settings, and seeking meaning and understanding.

By following a structured, hybrid analytic process [Figure 1] combining data mining, WAG modeling, and GPT-4–assisted labeling, this study demonstrates the effectiveness of computational qualitative methods in analyzing large-scale, unsolicited data on cyber victimization. This approach addressed the limitations of traditional qualitative research, especially in the context of handling large amounts of unstructured data. In addition, traditional qualitative research methods are often limited by participant selection biases and social desirability biases, which limit the breadth and depth of the narratives [10]. The anonymity of Reddit also encouraged users to share sensitive information without fear of stigma. This approach facilitated the identification of patterns and emerging themes that may not have been captured through manual coding alone.

This study provided a novel methodological approach for examining cyber victimization experiences and highlights the potential for AI-assisted qualitative analysis. GPT-4 was used in initial cluster labeling, which, when combined with manual review, improved the accuracy of thematic categorization. This builds on previous qualitative research by demonstrating how computational tools can be applied to analyze unsolicited narratives, reduce researcher bias, and identify themes across a vast dataset.

While hybrid computational-qualitative methods have been applied to topics such as substance abuse [13], epilepsy [14], and intimate partner violence [15], this study extends that work by applying similar techniques to cyber victimization. By doing so, it demonstrates the adaptability of WAG modeling and GPT-4–assisted labeling to new areas of public health. Recent studies support this hybrid approach. For example, Piper and Wu [33] found that large language models (LLMs) performed well in narrative topic labeling, while Castellanos et al [34] demonstrated that although GPT-4 generated themes aligned with human coding in over 79% of cases, human coders were still required for accuracy. Our integration of GPT-4–assisted labeling with manual review aligns with these findings and demonstrates the need for human oversight.

The findings have practical implications for health care settings. Recognizing the diverse ways users describe psychological impacts and coping strategies could inform the development of educational resources, screening instruments, and assessment strategies that reflect the language and experiences of victims. These resources would be valuable to health care professionals in identifying individuals at risk in primary care or mental health settings where cyber victimization may go unreported.

### Limitations

While this study has many strengths, it is not without limitations. Our data consisted of anonymous user narratives from Reddit, making it challenging to determine the generalizability of the sample to a wider population. Reddit users also may have specific characteristics, interests, or behaviors that are not reflective of a broader population [35]. In addition, social media platforms have distinct user demographics and cultures, which might influence the nature and extent of cyber victimization experienced by users. For example, Reddit users are more likely to be men [36,37], while TikTok (ByteDance Ltd), Facebook (Meta Platforms), and Pinterest (Pinterest, Inc) users are more likely to be women [37].

Due to the inability to follow up with users for explanation and clarification and the inability to meet users face-to-face to assess body language, vocal tones, and facial expressions, there was a potential for misinterpretation of context [38]. The anonymous nature of Reddit may also lead to false answers or misinformation, as internet-based platforms can be prone to exaggeration, false claims, or recall bias. In addition, demographic inferences cannot be made, as users are not required to disclose personal information.

An important limitation of this research is the potential inclusion of bot-generated content [39]. Despite efforts to identify and exclude bots based on patterns in posting behavior, timing, frequency, language use, and identifiable usernames, the sophisticated nature of some bots may have allowed them to bypass detection. It is possible that bot-generated posts, which do not reflect human experiences, were incorporated into the dataset, potentially influencing the results.

Another limitation relates to the evolving nature of LLMs. GPT-4, the model used at the time of this study (2023), had a significantly smaller context window, which limited the number of posts that could be processed per cluster. As a result, only a subset of cluster content was used for label identification. Newer versions of the model support much larger input sizes, which may produce different results, affecting replication in future studies.

Finally, while GPT-4 accelerated the qualitative analysis process, several limitations must be acknowledged. LLMs are prone to selective summarization and misrepresentation, known as hallucination [40]. LLMs may also simplify content while being overconfident in tone, which can influence researchers’ judgment by making inaccurate or biased content appear more credible than it is. These limitations may have affected the accuracy of cluster label identification. Manual validation was used to mitigate these risks. However, the reliance on GPT-4 for suggestive labeling remains a methodological limitation worth noting.

### Implications for Further Research

Future research could expand this methodology to further explore and deepen the understanding of cyber victimization experiences and further refine computational qualitative analysis techniques. While this study focused on cyber victimization experiences in 2 subreddits, other communities may provide further insight into cyber victimization. Future studies could extend data mining and WAG modeling to specific types of cyber victimization-related subreddits such as r/stalking, r/cyber security, and r/scams, which focus on distinct aspects of harmful experiences. Cyber victimization is broad; these specific subreddits could provide a more nuanced understanding of how different forms of cyber victimization are discussed within internet-based communities.

To explore how cyber victimization experiences vary across internet-based spaces with different user demographics and privacy structures, future research could compare narratives from different platforms (TikTok [ByteDance Ltd], Discord [Discord Inc], Bluesky [Bluesky PBLLC], Instagram [Meta Platforms], and Tumblr [Automattic]). Each platform has unique privacy settings, user demographics, and moderation policies, which may influence how users discuss and experience cyber victimization.

### Conclusions

This study used a hybrid methodological approach to analyze how users on Reddit describe their experience of cyber victimization using data mining and computational analysis of unsolicited data. By leveraging data mining and WAG modeling, this study demonstrated the effectiveness of computational methods in qualitative analysis. GPT-4-assisted labeling and focused keyword searching further refined thematic identification, resulting in 6 themes: psychological impact, coping and healing, protecting oneself online, protecting oneself offline, victimization across various settings, and seeking meaning and understanding. The methodological approach demonstrated in this study will be valuable to data scientists and health care researchers seeking to analyze social media data on mental health issues. These methods can inform future studies on risky internet behavior, victimization, and assessment strategies in health care settings.

## Supplementary material

10.2196/75493 Multimedia Appendix 1 Detailed methodological process for data mining.

10.2196/75493 Multimedia Appendix 2 Number of posts and comments per cluster.

10.2196/75493 Multimedia Appendix 3 List of cluster labels and thematic categorization.

10.2196/75493 Multimedia Appendix 4 Manual Review Outcomes for GPT-4 Generated Labels

10.2196/75493 Multimedia Appendix 5 Definitions and examples of GPT-4 label validation categories.

10.2196/75493 Multimedia Appendix 6 Example analysis and narrative linkages.

10.2196/75493 Multimedia Appendix 7 Summary of identified themes from thematic analysis.

## Abbreviations

AI: artificial intelligence
API: application programming interface
GPT-4: Generative pre-trained transformer
LLM: Large language model
SAMHSA: Substance Abuse and Mental Health Services Administration
WAG: Word Adjacency Graph
