# Supplementary material for: Data Mining Trauma: AI-Assisted Qualitative Study of Cyber Victimization on Reddit
Source: JMIR Infodemiology. 2025 Sep 3;5:e75493. doi: 10.2196/75493 (PMC12407219; doi:10.2196/75493)
Supplement: Multimedia Appendix 1 [file infodemiology-v5-e75493-s001.docx]

**Collection Requirements and Data Investigation**

Collecting requirements and data investigation involved defining the needs and the aim of the study and understanding Reddit's structure to identify challenges and limitations in data analysis [23]. This study identified the potential for irrelevant data, or "noise," to influence and potentially mislead the interpretation of results. Examples of noise include stop words such as "with," "the," and "a," which would not contribute meaningfully to the analysis [24].

Another concern was bot-generated content that does not reflect human experiences. Bot-generated content was filtered out by identifying patterns in posting behavior, such as frequency, standardized language, and bot-generated usernames. Bot-generated content refers to posts, comments, or other forms of content that are automatically generated and posted by software applications, known as bots, instead of human users [25-26].

**Data Collection**

Data were extracted using access to Reddit's application programming interface (API). Reddit's API grants researchers access to the platform's data, allowing for the extraction of user posts and comments. In addition, a custom web scraping tool was created and specifically designed to collect data relevant to the study from the r/cyberbullying and r/bullying subreddits. The search parameters were set to extract data from 2012-2023 with emphasis on the most upvoted posts within this time frame, as these posts were the most relevant and reflective of the community's experience. Access to the API was achieved in adherence to Reddit's API access rules.

**Modeling**

A member of our team (author D.G.) originally developed WAG modeling to identify relationships in text and focus on how words in a text connect and how frequently words and concepts appear near each other [16]. The WAG modeling process constructs a network graph in which words are represented as nodes in a network. Words that are adjacent to each other in the text form connecting edges, allowing for visualization of how words are interconnected, revealing patterns and relationships between different words and concepts that form clusters. These clusters identify common themes within the text [16]. Text from posts and comments are organized into word pairs after removing stop words. The resulting pairs of adjacent words are used to construct the nodes and edges of the WAG model, where nodes represent distinct words or phrases, and edges represent the adjacency relationship between nodes.

Building upon this foundation, this study applies WAG modeling to cyber victimization narratives on Reddit, leveraging the ability to reveal patterns in large sets of unstructured data. The Leiden algorithm [27], a mathematical method for detecting topics or themes in large networks, was used to arrange nodes into clusters. When the Leiden algorithm is applied to these data, it systematically organizes the text entries into clusters based on the frequency and patterns of word adjacency, identifying words and phrases that are commonly used in the context of each other and ultimately revealing underlying themes in the data.

**Assessment**

The nodes were manually reviewed in the assessment phase to identify relevance to the assigned cluster. Irrelevant topics assigned to a cluster were removed from the model; for example, the term "troll" might initially appear in a cluster due to its common association with harmful online behaviors. However, it may become clear that "troll" referred to fantasy creatures in a gaming context and was irrelevant to the cluster or cyber victimization. A sample of Reddit posts (10%) from each cluster was manually reviewed for relevance.

**Cluster Labeling**

GPT-4 was used to facilitate cluster labeling once the clusters were identified. GPT-4 is a large language model (LLM) developed by OpenAI [28]. In this study, its role was to summarize posts and comments and suggest potential labels for each cluster. A sample of posts from each cluster was selected for assessment. GPT-4 then generated suggested labels based on the sampled content. Following AI-assisted labeling, a manual review of the cluster data and their respective labels was conducted to ensure accuracy and relevance and to adjust where necessary.

**Keyword Searching**

To account for evolving language, we used focused keyword searching to capture variations, abbreviations, and related expressions across the entire data set. This approach ensured that relevant content was identified even if it did not fall neatly into clustered themes. MAXQDA's lemmatization feature identified word variants for semantically related terms [29]. For example, "thick skin" led to searches for similar phrases like "not taking things too seriously" or "brushing off comments." In addition, the study acknowledged the prevalence of abbreviations in online communication. To address this, the methodology included searches for common abbreviations that might signify cyber victimization. For example, the abbreviation "KYS" (kill yourself), often used in cyberbullying contexts, was specifically searched within the data set.

**Data Presentation**

Findings are presented through data visualization using Gephi, an open-source graph modeling tool for visualizing data [30]. Nodes and edges are color-coded and sized according to their weight, providing a clear and concise visual understanding of the trends and patterns related to cyber victimization. The results and the identified patterns and topics are discussed with examples of specific quotes for emphasis. Covert signs, symptoms, and narratives will be presented, linking to the original aim to analyze how users on Reddit describe and discuss their experience of cyber victimization.
