# Supplementary figures and images for: Data Mining Trauma: AI-Assisted Qualitative Study of Cyber Victimization on Reddit
Source: JMIR Infodemiology. 2025 Sep 3;5:e75493. doi: 10.2196/75493 (PMC12407219; doi:10.2196/75493)

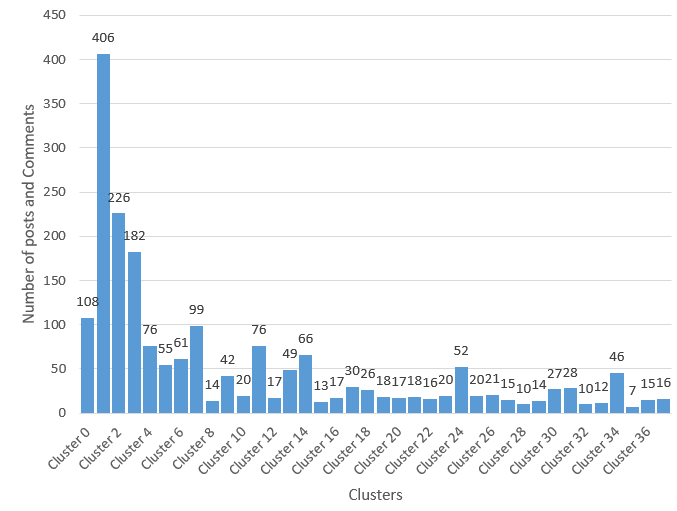

Supplement: Multimedia Appendix 2 [file infodemiology-v5-e75493-s002.png]
