# Supplementary material for: Data Mining Trauma: AI-Assisted Qualitative Study of Cyber Victimization on Reddit
Source: JMIR Infodemiology. 2025 Sep 3;5:e75493. doi: 10.2196/75493 (PMC12407219; doi:10.2196/75493)
Supplement: Multimedia Appendix 3 [file infodemiology-v5-e75493-s003.docx]

|  | | |  |
| --- | --- | --- | --- |
|  | | |  |
|  | GPT-4 Generated Labels | **Labels** | **Retained/ Adjusted/ Revised** |
| 0 | Stories of online bullying, harassment, and personal trauma | Long-term trauma and symptoms from cyber victimization | Adjusted |
| 1 | Victims share bullying experiences, seek help, and express pain and hope for recovery. | Advice from personal experience | Adjusted |
| 2 | Stories of cyberbullying, teachers’ indifference, and strategies to cope with bullying | Perceptions of school involvement and policy shortcomings | Adjusted |
| 3 | Workplace bullying defense coping strategies | Victimization in work and school | Adjusted |
| 4 | Personal stories reveal ongoing school bullying | School-linked long-term victimization trauma | Adjusted |
| 5 | Victims urged to contact police | Victims urged to seek legal recourse | Retained |
| 6 | Bullying impacts mental health | Mental health impact and therapy | Adjusted |
| 7 | Cyberbullying on social media incites depression and anxiety | Preventative strategies surrounding social media use. | Revised |
| 8 | Bullied victims seeking justice relief and healing | Self-help group therapy dynamics | Revised |
| 9 | Bullying within families | Impact of family | Revised |
| 10 | Overcoming bullying, moving forward | Overcoming victimization | Retained |
| 11 | Online bullying pain | Responses and resilience to victimization | Revised |
| 12 | Trauma from bullying triggers panic attacks | Past trauma triggers panic attacks | Retained |
| 13 | Persistent cyberbullying lack of intervention | Persistent victimization, feelings of loss of control | Adjusted |
| 14 | Blaming victims undermines anti-bullying efforts | Victim blame and dismissal experiences | Adjusted |
| 15 | Victims grapple with bullying seeking help; others downplay its significance. | Victims grapple with seeking help; others downplay the significance | Retained |
| 16 | Eye contact, significance, and overcoming it | Concept of eye contact in avoiding victimization | Adjusted |
| 17 | Become less susceptible to bullying | Becoming less susceptible to victimization | Retained |
| 18 | Victims use gaming for escapism | Support and strategies for addressing unwanted video/photo sharing | Revised |
| 19 | Victims struggle with unaddressed sexual harassment | Victims struggle with unaddressed sexual harassment | Retained |
| 20 | Data tracking harassers by ip address | Users seek help to determine aggressor identities | Adjusted |
| 21 | Seeking justice through law enforcement | Seeking justice through legal recourses | Merged/Retained |
| 22 | Legal action against bullying | Legal action against victimization | Merged/Retained |
| 23 | Warning bullies of spiritual and social consequences | Warning bullies of spiritual and social consequences | Excluded: Cluster, not relevant |
| 24 | Martial arts boosts confidence against bullies | Martial arts as a tool for confidence and self-defense | Retained |
| 25 | Filing restraining order seek harassment | Restraining order discussions and advice | Adjusted |
| 26 | Ignoring bullies to diminish their power | Ignoring to diminish aggressor power \| influence | Retained |
| 27 | Lifelong psychological effects of bullying | Exploring aggressor and victim dynamics. Seeking understanding and resources | Revised |
| 28 | Being a punching bag, reactions ranging from retaliation to despair | Punching bag versus self-advocacy | Adjusted |
| 29 | Coping mechanisms and impacts of cyberbullying | Coping strategies | Retained |
| 30 | Trauma from bullying, struggle to cope | Persistent effects of unaddressed victimization: | Revised |
| 31 | Rejecting thick skin as a solution to bullying | Rejecting thick skin as a solution to victimization | Retained |
| 32 | Cyberbullying is rampant in small communities | Small-town dynamics in victimization | Revised |
| 33 | Stand your ground against bullies. | Standing your ground | Retained |
| 34 | Overcome bullying, stay strong, seek support | Instilling hope and resilience | Revised |
| 35 | Bullying leads to suicidal thoughts | Victimization leads to suicidal thoughts | Retained |
| 36 | Bullying viewed from various perspectives | Victimization viewed from various perspectives | Excluded: Cluster not relevant |
| 37 | Bullying causes long-term effects | User theories on aggression and victimization | Revised |
