# Supplementary material for: Data Mining Trauma: AI-Assisted Qualitative Study of Cyber Victimization on Reddit
Source: JMIR Infodemiology. 2025 Sep 3;5:e75493. doi: 10.2196/75493 (PMC12407219; doi:10.2196/75493)
Supplement: Multimedia Appendix 4 [file infodemiology-v5-e75493-s004.docx]

|  |  |
| --- | --- |
|  |  |
| Theme | **Summary** |
| Psychological and emotional impact | - Severity of impact on mental health - Vivid descriptions of distress. - Overlap between traditional victimization and cyber victimization |
| Coping and healing | - Concerns over placing responsibility on victims - Importance of support systems - Need for online spaces |
| Protecting yourself online | - Seeking practical help - Identity theft - Legal options - Seeking justice |
| Protecting yourself online | - Reducing vulnerability - Need for others to intervene |
| Victimization across various settings | - Failure of authority to address the problem - Perception of environments as hostile - Victimization seen as inevitable |
| Seeking meaning and understanding | - Discussions surrounding theories of victimization - Attempts to make sense of experiences - Reflections on the causes of victimization |
