# Supplementary material for: Data Mining Trauma: AI-Assisted Qualitative Study of Cyber Victimization on Reddit
Source: JMIR Infodemiology. 2025 Sep 3;5:e75493. doi: 10.2196/75493 (PMC12407219; doi:10.2196/75493)
Supplement: Multimedia Appendix 5 [file infodemiology-v5-e75493-s005.docx]

|  | |  |
| --- | --- | --- |
|  | |  |
| Category | **Definition** | **Example Cluster #** |
| Retained | Closely matched the cluster content and required no changes or only minor edits. | **Cluster 5:** "Victims urged to contact police" was retained as "Victims urged to seek legal recourse."  **Cluster 10:** "Overcoming bullying, moving forward” was retained as “Overcoming victimization." |
| Adjusted | Generally aligned with the cluster’s theme but required moderate rewording to enhance clarity, specificity, or tone. | **Cluster 1:** "Victims share bullying experiences, seek help, and express pain and hope for recovery" was adjusted to "Advice from personal experience."  **Cluster 6:** "Bullying impacts mental health" was adjusted to "Mental health impact and therapy." |
| Revised | Did not reflect the content and was replaced entirely by the researcher. | **Cluster 8:** "Bullied victims seeking justice relief and healing" was revised to "Self-help group therapy dynamics."  **Cluster 30:** "Trauma from bullying, struggle to cope" was revised to "Persistent effects of unaddressed victimization." |
| Merged | clusters with substantial thematic overlap were combined under a single refined label. | **Clusters 21 & 22:** "Seeking justice through law enforcement" and "Legal action against bullying" were merged into "Legal action against victimization." |
| Excluded | The cluster was removed from analysis due to off-topic, irrelevant, or spam-like content. | **Cluster 23:** "Warning bullies of spiritual and social consequences" was excluded as not relevant.  **Cluster 36:** "Bullying viewed from various perspectives" was excluded due to lack of thematic coherence. |
