# Supplementary material for: Data Mining Trauma: AI-Assisted Qualitative Study of Cyber Victimization on Reddit
Source: JMIR Infodemiology. 2025 Sep 3;5:e75493. doi: 10.2196/75493 (PMC12407219; doi:10.2196/75493)
Supplement: Multimedia Appendix 6 [file infodemiology-v5-e75493-s006.docx]

|  |  |  |  |
| --- | --- | --- | --- |
| Example Narrative | **Alterative Expression** | **Focused searches** | **Similar Experiences in narrative outside the cluster** |
| "He tells me he still has all the photos and he plans on keeping them to look at and to send to my family and friends. " | blackmail, catfish | "blackmail" "catfish" | **"**Need advice about […] blackmail) ... he took some pics / video of her [...] now he is threatening to publish them on social media**"** |
| "I have been traumatized by this topic to the point of asking my mother every day and every night if I am beautiful and if I deserve to live just because some idiots told me every day that I deserve to die, no one likes me and that I deserve the abuse" | intrusive thoughts  unable to stop thinking about abuse | "stop thinking" | **"**I can't stop thinking about the way this kid made me feel … I couldn't defend myself I just sat there and took it. I think about it all the time the shame and anger and inferiority.**"** |
| "They made contact with me 3 months after the falling out and started doxing me, They have personal videos/pictures of myself. They have made Tik tok accounts, dating sites using my government name" | invasion of privacy  identity theft | "dox"  "privacy" "identity" | **"**Someone doxxed my entire life story on instagram-full name, where i live, where i used to work, where i go to college, even relatives (named one who is deceased) … I am however very creeped out and angry.**"** |
| "And hammer yourself down so you don't stick out." | blending in  conforming  staying under the radar | "blend"  "conform"  "radar" | **"**I keep to myself, don’t stare at people and do my best to just blend in, but it never seems to work.**"** |
